# Experimental realization of ultrathin, double-sided metamaterial perfect absorber at terahertz gap through stochastic design process

**DOI:** 10.1038/srep18605

**Published:** 2015-12-22

**Authors:** Tsung-Yu Huang, Ching-Wei Tseng, Ting-Tso Yeh, Tien-Tien Yeh, Chih-Wei Luo, Tahsin Akalin, Ta-Jen Yen

**Affiliations:** 1 Department of Materials Science and Engineering, National Tsing Hua University, Hsinchu, Taiwan, R.O.C; 2 Department of Materials Science Center for Nanotechnology, Materials Science, and Microsystems, National Tsing Hua University, Hsinchu, Taiwan, R.O.C; 3 Department of Electrophysics, National Chiao Tung University, Hsinchu, Taiwan, R.O.C; 4 Institute of Electronic, Microelectronic and Nanotechnology, Lille University, France

## Abstract

We design and demonstrate a flexible, ultrathin and double-sided metamaterial perfect absorber (MPA) at 2.39 terahertz (THz), which enables excellent light absorbance under incidences from two opposite sides. Herein, the MPA is fabricated on a λ_0_/10.1-thick flexible polyethylene terephthalate substrate of ε_r_ = 2.75 × (1 + 0.12*i*), sandwiched by two identical randomized metallic patterns by our stochastic design process. Such an MPA provides tailored permittivity and permeability to approach the impedance of free space for minimizing reflectance and a great imaginary part of the refractive index for reducing transmittance and finally results in high absorbance. Both experimental measurement and numerical simulation are in a good agreement. The flexible, ultrathin and double-sided MPA significantly differs from traditional quarter-wavelength absorbers and other single-sided perfect absorbers, paving a way toward practical THz applications in thermal emission, sensing and imaging, communications, stealth technique, and even energy harvesting.

First introduced by W. J. Padilla *et al.* in 2008^1^, metamaterial-based perfect absorbers (MPAs) have soon attracted researchers’ attention due to their abilities to significantly suppress the thickness of the absorbers into a sub-wavelength scale, λ_0_/35 and λ_0_/33 (excluding 500-μm-thick GaAs substrate) at microwave and terahertz regions^1^,^2^, respectively, in contrast to the traditional Salisbury or Jaumann screens with the thicknesses of quarter-wavelengths for single or multiple radar frequencies. An MPA is typically comprised of two distinct metallic layers with a dielectric spacer. There correspond two keys to design these two metallic layers– one is to design the first metallic layer and then tailor constitutive parameters (i.e., ε and μ) of entire unit cell to approach free space impedance for minimizing reflectance (R); the other is to employ a back reflector as the second metallic layer, such as a plasmonic line^2^ or a metallic ground plane^3^,^4^,^5^,^6^,^7^, for blocking transmittance (T). Once both of reflectance and transmittance are minimized, one can certainly maximize the absorbance (A; A = 1-R-T). Based on these two keys, recently several MPAs have been demonstrated, such as broadband^3^,^4^,^5^, wide-angle^6^ and polarization insensitive^7^ absorbers. Yet, the employment of the back reflector essentially sets a constraint on the incident direction into the single one. For example, if waves impinge from the side of the back reflector, then the wave will not be absorbed but reflected by this MPA. In this case, a perfect absorber suddenly becomes a perfect reflector, which plagues the practical applications.

More recently, an alternative design termed as metasurfaces has been reported to absorb light^8^,^9^,^10^. By introducing a phase shift from the metasurfaces, the multi-reflected waves destructively interfere with each other, reducing a reflectance down to 0.25%, and the transmitted wave is completely absorbed by adjusting the optical loss of the optical thin film. Still, this kind of metasurfaces encounters the same limitation of single-sided applications, like conventional MPAs do. Another route to absorbing light can be achieved by means of transformation optics to construct an optical black hole^11^,^12^,^13^. Unfortunately, this optical black hole is especially bulky under grazing angle incidence. As a consequence, to address the challenges abovementioned, in this work we introduce a flexible, ultrathin and double-sided MPA at the terahertz (THz) gap on the basis of the effective medium theory^14^,^15^. Through our developed computer-aided stochastic design process, we can efficiently acquire patterns with the absorbance close to unity under bi-directional incidences, yielding practical applications including thermal emitters^16^, focal planar array imaging^17^,^18^, plasmonic sensors^19^, communication devices^20^, stealth materials^21^ and even energy harvesting materials^22^.

## Results

### Patterns of double-sided metamaterial perfect absorber from stochastic design process

**Figure 1:**
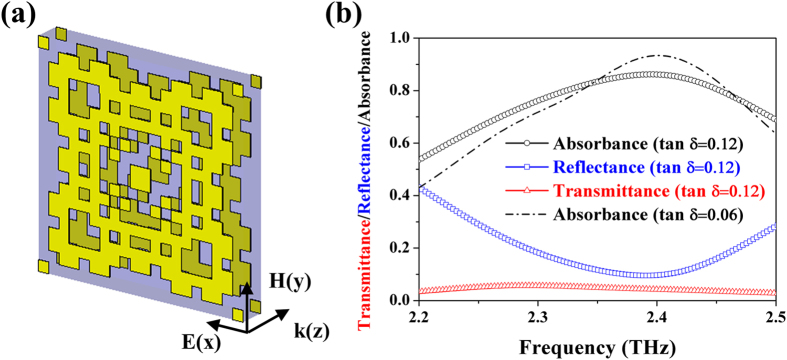
A double-sided metamaterial perfect absorber (MPA) and its performance. (**a**) A schematic view of a double-sided MPA with metallic patterns developed through a stochastic process. In simulation, we set the unit cell boundary conditions in the both x and y-directions to emit a plane wave with the polarization oriented in the x direction. (**b**) Simulated reflectance (blue symbols), transmittance (red symbols), and absorbance (black symbols) of the double-sided MPA. The substrate is a flexible polyethylene terephthalate (PET) thin film of 12-μm-thick, with loss tangent (tan δ) of 0.12. The corresponding reflectance and transmittance are 9.5% and 4.3%, respectively, resulting in a remarkable absorbance of 86.2% at 2.39 THz. One can further intensify the absorbance by employing a less lossy PET film (e.g., 93.4% for tan δ = 0.06).

The double-sided MPA is comprised of a metal-insulator-metal (MIM) structure. For the insulating substrate, we utilized a flexible polyethylene terephthalate (PET) thin film with the electric permittivity (ε_r_) of 2.75 × (1 + 0.12*i*) and the thickness of 12 μm, about λ_0_/10.1-thick. As for the metallic pattern of the double-sided MPA, it is worth mentioning that it is not achievable by simply copying the front pattern to replace the back-reflector in cases of conventional MPAs^23^,^24^. To conquer this obstacle, we develop a stochastic process to design the metallic patterns on the two sides. In this stochastic process, the metallic patterns are developed through discretizing the unit cell into 20 × 20 pixels with the pixel size of 5 × 5 μm^2^ (i.e., unit cell size equal to 100 × 100 μm^2^). In addition, we only consider the one eighth of the metallic pattern in the unit cell to create a four-fold symmetric pattern by mirroring the generated patterns three times to achieve the polarization-insensitive performance. Then, we randomized a value between 0 and 1 to determine whether or not there exists metal on a pixel (see Method for more details). By repeating this random process on each pixel, we could obtain the metallic patterns capable of double-sided absorbance. Note that the stochastic design process could be the very first step of the genetic algorithm, thus possessing a potential to further optimize the performance of the MPA through targeted goals in a genetic algorithm^25^,^26^,^27^,^28^. Finally, among two thousand stochastic patterns, the best design of the double-sided MPA is presented in Fig. 1(a). To testify the performance of our double-sided MPA, we employed a finite-integration simulation method, *CST Microwave Studio*^*TM*^, to numerically calculate the corresponding reflectance, transmittance and absorbance. In simulation, we choose build-in material properties of gold with conductivity of 4.56 × 10^7^ S/m, which is sufficient for simulation within terahertz region^7^. As shown in Fig. 1(b), the reflectance and transmittance equal to 9.5% and 5.3% at 2.39 THz, respectively, indeed leading to an absorbance peak of 86.2% within the THz gap. More importantly, the absorbance would further elevate up to 93.4% as shown in Fig. 1(b) once a PET film with less loss tangent (tan δ), for example, equal to 0.06 is available for the experiment (see Fig. S1 and Fig. S2 in Supplementary Information for further discussion).

### Surface currents and field distribution

**Figure 2:**
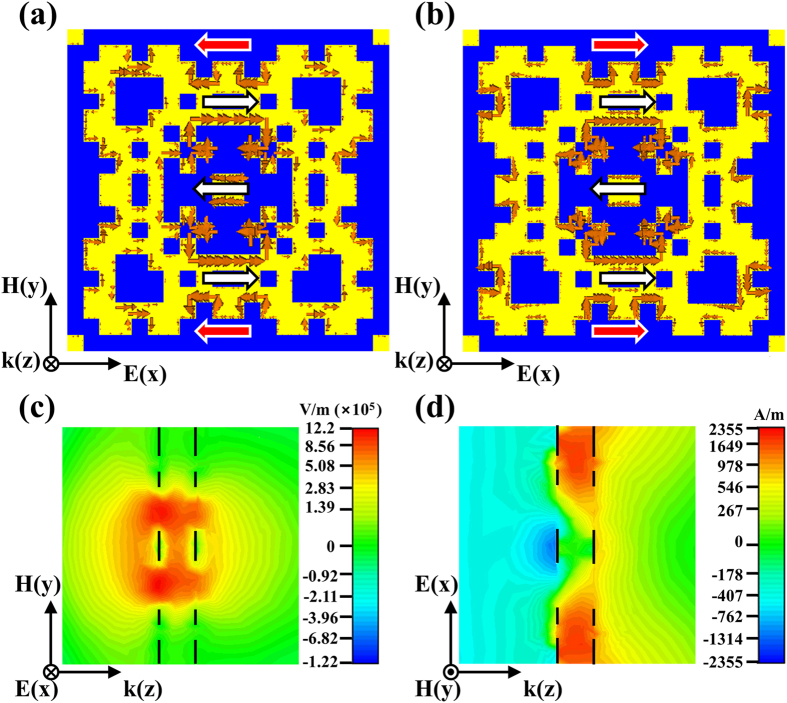
Surface current and field distributions of the double-sided MPA. Top views of the surface current distribution (**a**) on the first metallic patterns and (**b**) on the second metallic pattern. The currents on the first and second metallic patterns exhibit a pair of anti-parallel currents (denoted by the red arrows) resulting in a magnetic response at the certain area. On the other hand, the parallel surface currents on each surface demonstrate an electric response (indicated by white arrows). Side views of the (**c**) electric field and (**d**) magnetic field distributions present localized fields concentrating mainly within the PET and near the metallic patterns.

The mechanism of the double-sided MPA can be clarified by scrutinizing the current distributions within the metallic pattern. As shown in Fig. 2(a,b), there exist the parallel currents, flowing within each metallic pattern (labeled by the white arrows on each side). These parallel currents support the excitation of the electric dipoles, modulating the effective electric permittivity; meanwhile, there also appear anti-parallel currents flowing between two metallic layers (labeled by the red arrows), which enable the excitations of the magnetic dipoles, giving rise to the demanded magnetic permeability in the nonmagnetic materials to balance the impedance between the MPA and free space. Moreover, we observe that both of the electric and magnetic fields are mainly localized within the dielectric layer and few adjacent to the metallic patterns, indicated in Fig. 2(c,d). Such localized electromagnetic fields dissipating in the spacer might benefit the field of energy harvesting when PET is replaced by semiconductor^29^,^30^ (see Fig. S3 in Supplementary Information for more details).

### Retrieval data of complex electric permittivity, magnetic permeability and refractive index

**Figure 3:**
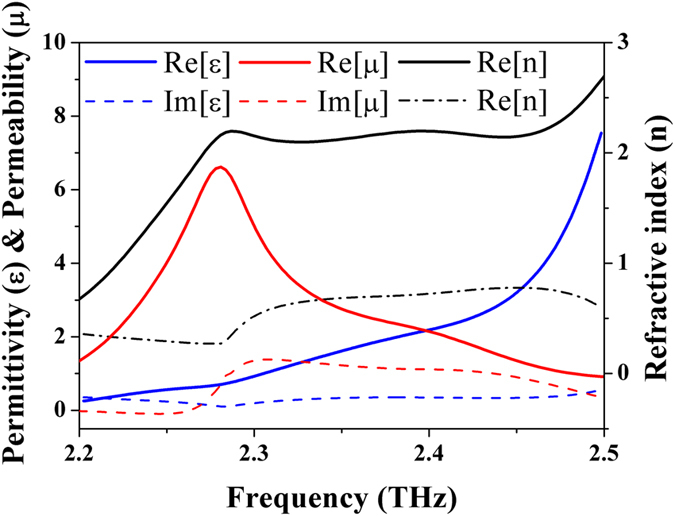
Retrieval data of the double-sided MPA. The retrieval data implies a matching impedance and great imaginary part of index leading to minimum reflectance and transmittance, respectively at the frequency of absorbance maximum, i.e., 2.39 THz.

To further comprehend the mechanism of the double-sided MPA, we employed the conventional sophisticated retrieval method to extract the complex permittivity, permeability and effective refractive index, respectively^14^,^15^. The real part of permittivity and permeability are intersected around 2.39 THz as shown in Fig. 3, resulting in a minimized reflectance by matching the impedance to the one of free space. Moreover, the imaginary part of the index presents an enhanced value of 0.72, which is almost 7 times lager compared to the intrinsic losses of the PET film itself; thus, the proposed double-sided MPA could absorb the incident energy and then give rise to low transmittance. Therefore, according to the retrieval data, suggesting the matched impedance and enhanced imaginary part of the index, we could predict an absorption peak occurs at 2.39 THz in the double-sided MPA, which is consistent with the simulated spectrum as portrayed in Fig. 1(b). It is worthy stating that we also employ the multiple reflection theory to explain the behavior of our proposed perfect absorber as suggested in ref. 24, but we can only achieve a similar response after irrationally increasing the corresponding losses of the substrate by 100 times; moreover, the reduction of reflection stems from the matched impedance rather than the destructive interference suggested by the multiple reflection theory due to the fact that the direct reflection itself is small enough compared to the overall reflection with minor influences from the consecutive multiple reflection. Thus, we claim that the enabling factor of our double-sided MPA originated from the matching impedance and great effective propagating losses of the spacer evidenced by the effective medium theory instead of the multiple interference theory.

### Sample fabrication and characterization

**Figure 4:**
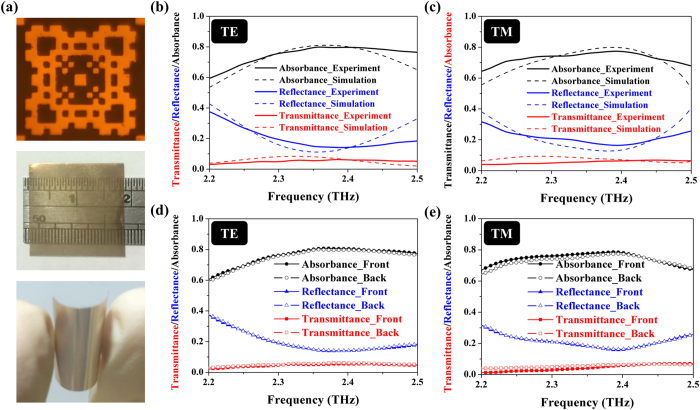
Experiment results of the double-sided MPA. (**a**) Optical microscope image of the unit cell, the photograph of 18 × 18 mm^2^ fabricated samples and the bent sample showing the flexibility. Clearly, the doubled-sided PA is transparent at the visible range. Experimental (solid line) and simulation (dashed line) results of the double-sided PA in (**b**) TE and (**c**) TM incident conditions. The two reveal an excellent agreement. Moreover, the double-sided absorption is demonstrated by the experiments at both the front-sided (solid symbols) and back-sided (hollow shape) incidences for (**d**) TE and (**e**) TM cases as well.

For the sample fabrication, we applied an ultraviolet (UV) lithography technique and an electron-gun deposition process, and then a lift-off technique to realizing the designed metallic pattern. Since both of the rotation and translation alignment between the two metallic patterns of the double-sided MPA are a request to ensure the functionality of the structure, we also employed an alignment mark to provide 2-μm accuracy. The fabricated structure is comprised of 10-nm-thick titanium (as an adhesion layer) and 200-nm-thick gold films, and its unit cell is shown in the upper part of Fig. 4(a). Besides, we adopted a commercially available PET film as a dielectric spacer. This flexible PET film is 12-μm-thick, about a tenth of the working wavelength with a complex dielectric constant ε_r_ = 2.75 × (1 + 0.12*i*). Finally, a flexible, ultrathin, optically transparent and double-sided MPA is presented in the middle and lower parts of Fig. 4(a).

Next, we characterized the performance of the fabricated MPA by a micro-Fourier transform infrared (μ-FTIR) spectrometer (Vertex 70V) in the frequency range from 2.2 to 2.5 THz. Notice that we conducted the reflectance measurement at 11° instead of normal incidence, and in this case, there appear two different conditions of TE and TM incidences. As shown in Fig. 4(b,c), the measured absorbance is up to 78.6% for the TE incidence and 80.8% for the TM incidence, respectively. Such similar values of absorbance for two different incidence cases reflect that the MPA is insensitive to polarization due to its 4-fold symmetric design. Herein a great portion of loss originates from the PET film, so that one can enhance the absorbance over 90% as predicted in the simulation (see Fig. 1) by simply using a more transparent film. In addition, we also characterized the performance of the MPA, under the incidence from the other side. The corresponding results are shown in Fig. 4(d,e). As we expected, these results are similar to prior Fig. 4(b,c), indicating that this MPA is indeed a double-sided device. Note that all the results shown in Fig. 4(b–e), including the experiment (solid lines) and the simulation (dashed line) are in an excellent agreement. The little offset between the experiment and the simulation results from the fabrication imperfection, mainly caused by the uncontrollable flatness of the 12-μm-thin and flexible PET substrate.

Finally, the fabricated double-sided MPA is ultrathin, with sub-wavelength thickness of λ_0_/10.1, where λ_0_ is the free space wavelength. In fact, we could further reduce the thickness of the double-sided MPA once we obtain a thinner substrate in stock (see Fig. S4 and Fig. S5 in Supplementary Information for discussion of thickness- and angle-dependence of our double-sided MPA).

## Discussion

In conclusion, we have stochastically developed metamaterial patterns to successfully demonstrate an MPA within the THz gap. At 2.39 THz, we demonstrated remarkable absorbance up to 80.8%, under the 11° off-normal incidence in μ-FTIR measurement. The enabling factors of this MPA are minimizing the reflectance by approaching impedance-matching between free space and the MPA, and diminishing the transmittance by maximizing effective imaginary part of refractive index of the MPA, respectively. Different from other absorbers, the demonstrated sample can be operated for both-side applications, and is flexible and ultrathin with the thickness of λ_0_/10.1 that can be further miniaturized by employing a thinner flexible thin film. In addition, the field distributions within the metallic pattern and the dielectric spacer of the MPA indicate that the major energy is absorbed within the dielectric layer, instead of dissipated as heat in the metallic pattern. Therefore, not only this absorber can be readily employed for thermal emission, sensing/imaging, communications and stealth technique, but it could also facilitate harvesting photon energy.

## Methods

### Stochastic design process

In the first step of the stochastic design process, a unit cell of the double-sided metamaterial perfect absorber (MPA) is divided into 20 × 20 square pixels and then whether there would appear a metallic pattern on the front and back sides of each pixel or not depends on a randomized number between 0 to 1. Once the random number is smaller than 0.5, there would not appear metal on that pixel; otherwise, there would exist metallic squares on both sides. Further, in order to achieve a polarization-independent pattern, we only encode one eighth of total 400 pixels and generate a four-fold symmetric pattern by mirroring the one-eighth pattern three times. Thus, by repeating the random process on the one-eighth pixels 55 times, we can easily achieve a stochastic designed double-sided MPA whose performance is further determined in the aid of the commercial electromagnetic solver of the finite integration method.

### Sample fabrication

1. Designed photo-mask of metamaterial pattern from stochastic process
2. Attached 12-μm-thick PET on Si substrate
3. Conducted UV photolithography
4. Deposited 10 nm-thick titanium and 200 nm-thick gold
5. Proceeded Lift-off process
6. Flipped the 12 μm-thick PET with first pattern
7. Repeated step1–6 for the second pattern with tolerable alignment on the other side

### Measurement

Micro-Fourier transform infrared (μ-FTIR) spectrometer (Vertex 70V)

Light source: Mercury lamp

Spectral range: 100–10 cm^−1^

Spectral resolution: 1 cm^−1^

Aperture size: 8 mm

Scan velocity: 1.6 kHz

## Additional Information

**How to cite this article**: Huang, T.-Y. *et al.* Experimental realization of ultrathin, double-sided metamaterial perfect absorber at terahertz gap through stochastic design process. *Sci. Rep.* **5**, 18605; doi: 10.1038/srep18605 (2015).

## Supplementary Material

Supplementary Information

## References

[b1] Landy N. I., Sajuyigbe S., Mock J. J., Smith D. R. & Padilla W. J. Perfect Metamaterial Absorber. Phys. Rev. Lett. 100, 207402 (2008). 18518577 10.1103/PhysRevLett.100.207402

[b2] Tao H. *et al.* A metamaterial absorber for the terahertz regime: Design, fabrication and characterization. Optics Express 16, 7181–7188 (2008). 18545422 10.1364/oe.16.007181

[b3] Wakatsuchi H., Greedy S., Christopoulos C. & Paul J. Customised broadband metamaterial absorbers for arbitrary polarization. Optics Express 18, 22187–22198 (2010). 20941120 10.1364/OE.18.022187

[b4] Aydin k., Ferry V. E., Briggs R. M. & Atwater H. A. Broadband polarization-independent resonant light absorption using ultrathin plasmonic super absorbers. Nature Communications 2, 517 (2011). 10.1038/ncomms1528 22044996

[b5] Sun J., Liu L., Dong G. & Zhou J. An extremely broad band metamaterial absorber based on destructive interference. Optics Express 19, 21155–21162 (2011). 22108966 10.1364/OE.19.021155

[b6] Pu M. *et al.* Design principles for infrared wide-angle perfect absorber based on plasmonic structure. Optics Express 19, 17413–17420 (2011). 21935107 10.1364/OE.19.017413

[b7] Grant J. *et al.* Polarization insensitive terahertz metamaterial absorber. Optics Letters 36, 1524–1526 (2011). 21499411 10.1364/OL.36.001524

[b8] Kats M. A., Blanchard R., Genevet P. & Capasso F. Nanometre optical coatings based on strong interference effects in highly absorbing media. Nature Materials 12, 20–24 (2012). 23064496 10.1038/nmat3443

[b9] Dotan H. *et al.* Resonant light trapping in ultrathin films for water splitting. Nature Materials 12, 158–164 (2012). 23142836 10.1038/nmat3477

[b10] Yu N. & Capasso F. Flat optics with designer metasurfaces. Nature Materials 13, 139–150 (2014). 24452357 10.1038/nmat3839

[b11] Narimanov E. E. & Kildishev A. V. Optical black hole: Broadband omnidirectional light absorber. Appl. Phys. Lett. 95, 041106 (2009).

[b12] Genov D. A., Zhang S. & Zhang X. Mimicking celestial mechanics in metamaterials. Nature Phys. 5, 687–692 (2009).

[b13] Sheng C., Liu H., Wang Y., Zhu S. N. & Genov D. A. Trapping light by mimicking gravitational lensing. Nature Photonics 7, 902–906 (2013).

[b14] Smith D. R., Vier D. C., Koschny T. & Soukoulis C. M. Electromagnetic parameter retrieval from inhomogeneous metamaterials. Phys. Rev. E 71, 036617 (2005). 10.1103/PhysRevE.71.036617 15903615

[b15] Liu X. X., Powell D. A. & Alù A. Correcting the Fabry-Perot artifacts in metamaterial retrieval procedures. Phys. Rev. B 84, 235106 (2011).

[b16] Diem M., Koschny T. & Soukoulis C. M. Wide-angle perfect absorber/thermal emitter in the terahertz regime. Phys. Rev. B 79, 033101 (2009).

[b17] Liu X., Starr T., Starr A. F. & Padilla W. J. Infrared Spatial and Frequency Selective Metamaterial with Near-Unity Absorbance. Phys. Rev. Lett. 104, 207403 (2010). 20867064 10.1103/PhysRevLett.104.207403

[b18] Kuznetsov S. A., Paulish A. G., Gelfand A. V., Lazorskiy P. A. & Fedorinin V. N. MATRIX STRUCTURE OF METAMATERIAL ABSORBERS FOR MULTISPECTRAL TERAHERTZ IMAGING. Prog. Electromagn. Res. 122, 93–103 (2012).

[b19] Liu N., Mesch M., Weiss T., Hentschel M. & Giessen H. Infrared Perfect Absorber and Its Application As Plasmonic Sensor. Nano Lett. 10, 2342–2348 (2010). 20560590 10.1021/nl9041033

[b20] Watts C. M., Liu X. & Padilla W. J. Metamaterial electromagnetic wave absorbers. Adv. Mater. 24, 98–120 (2012). 10.1002/adma.201200674 22627995

[b21] Iwaszczuk K. *et al.* Flexible metamaterial absorbers for stealth applications at terahertz frequencies. Optics Express 20, 635–643 (2012). 22274387 10.1364/OE.20.000635

[b22] Wang H. *et al.* Highly efficient selective metamaterial absorber for high-temperature solar thermal energy harvesting. Solar Energy Materials & Solar Cells 137, 235–242 (2015).

[b23] Imhof C. & Zengerle R. Experimental verification of negative refraction in a double cross metamaterial. Appl. Phys. A 94, 45–49 (2008).

[b24] Chen H. T. Interference theory of metamaterial perfect absorbers. Optics Express 20, 7165–7172 (2012). 22453398 10.1364/OE.20.007165

[b25] Kern D. J. & Werner D. H. A GENETIC ALGORITHM APPROACH TO THE DESIGN OF ULTRA-THIN ELECTROMAGNETIC BANDGAP ABSORBERS. Microw. Opt. Technol. Lett. 38, 61–64 (2003).

[b26] Chen P. Y., Chen C. H., Wang H., Tsai J. H. & Ni W. X. Synthesis design of artificial magnetic metamaterials using a genetic algorithm. Optics Express 16, 12806–12818 (2008). 18711520 10.1364/oe.16.012806

[b27] Iwanaga M. Optically deep asymmetric one-dimensional plasmonic crystal slabs: Genetic algorithm approach. J. Opt. Soc. Am. B 26, 1111–1118 (2009).

[b28] Feichtner T., Selig O., Kiunke M. & Hecht B. Evolutionary Optimization of Optical Antennas. Phys. Rev. Lett. 109, 127701 (2012). 23005987 10.1103/PhysRevLett.109.127701

[b29] Atwater H. A. & Polman A. Plasmonics for improved photovoltaic devices. Nature Materials 9, 205–213 (2010). 20168344 10.1038/nmat2629

[b30] Wang Y. *et al.* Metamaterial-Plasmonic Absorber Structure for High Efficiency Amorphous Silicon Solar Cells. Nano Lett. 12, 440–445 (2012). 22185407 10.1021/nl203763k

