# Supplementary material for: Experimental realization of ultrathin, double-sided metamaterial perfect absorber at terahertz gap through stochastic design process
Source: Sci Rep. 2015 Dec 22;5:18605. doi: 10.1038/srep18605 (PMC4686993; doi:10.1038/srep18605)
Supplement: Supplementary Information [file srep18605-s1.pdf]

Experimental realization of ultrathin, double-sided metamaterial perfect absorber at terahertz gap through stochastic design process

Tsung-Yu Huang[1,+], Ching-Wei Tseng[1,+], Ting-Tso Yeh[1] , Tien-Tien Yeh[3], Chih-Wei Luo[3], Tahsin Akalin[4], and Ta-Jen Yen[1,2,*]

[1]Department of Material Science and Engineering, National Tsing Hua University, Hsinchu, Taiwan, R.O.C.

[2]Department of Materials Science Center for Nanotechnology, Materials Science, and Microsystems, National Tsing Hua University, Hsinchu, Taiwan, R.O.C.

[3]Department of Electrophysics, National Chiao Tung University, Hsinchu, Taiwan, R.O.C.

[4]Institute of Electronic, Microelectronic and Nanotechnology, Lille University, France

[*]tjyen@mx.nthu.edu.tw

[+]These two authors contributed to this work equally

- **Influences from a polyethylene terephthalate (PET) substrate with different losses and broadband responses of double-sided metamaterial perfect absorber (MPA)**

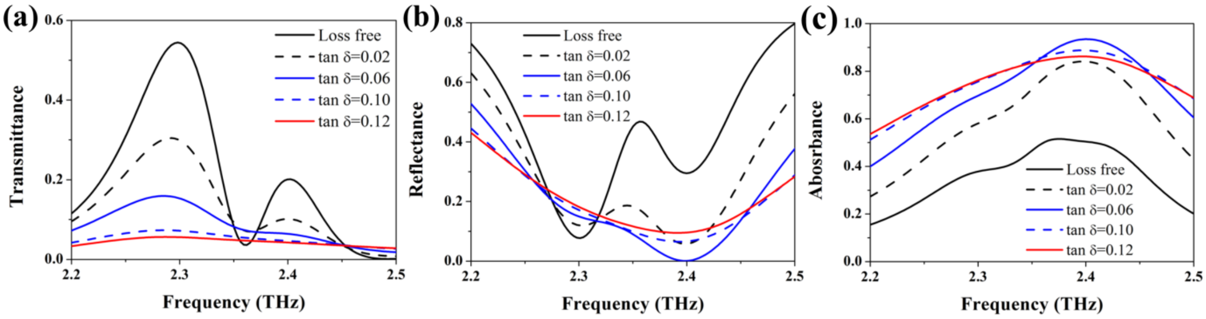

Figure S1. Simulated (a) transmittance, (b) reflectance and (c) absorbance of the double-sided metamaterial perfect absorber (MPA) with varied loss tangent of polyethylene terephthalate (PET).

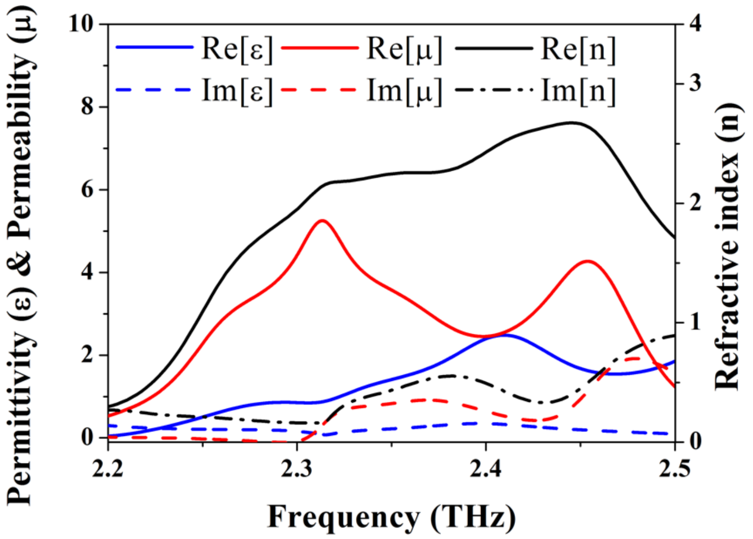

Figure S2. Retrieved constitutive parameters of the MPA with tan δ = 0.06. We could observe a smaller difference between imaginary parts of permittivity and permeability, thus resulting in a smaller reflectance and then higher absorbance.

To realize influences from loss tangent of a substrate, we plot the corresponding changes of transmittance, reflectance and absorbance as well with varied tan δ from 0 to 0.12 as portrayed in Fig. S1(a), S1(b) and S1(c), respectively. From these figures, we focus on the frequency of 2.4 THz where the absorbance reaches its maximum. In Fig. S2(a), it is clear that transmittance gradually decreases with increasing values of loss tangent and saturates at

around tan δ = 0.10. Besides, the transmittance difference is in the range of 65% (i.e., $\Delta T = \frac{T_{loss\,free} - T_{0.06}}{T_{0.06}}$); nevertheless, in Fig. S2(b), the reflectance does not exhibit such monotonic trend with respect to the varied tan δ. More importantly, the reflectance difference is up to 30200% (i.e., $\Delta R = \frac{R_{loss\,free} - R_{0.06}}{R_{0.06}}$), indicating that the reflectance substantially influences the absorbance (i.e., A=1-T-R), as shown in Fig. S2(c). Such different reflectance could be attributed to the different ratio of permeability and permittivity, i.e., the wave impedance of the MPA. Figure S2 shows a retrieval result of an MPA with tan δ = 0.06 revealing that such an MPA possess an excellent matching of wave impedance (1.018+0.047i for tan δ = 0.06; 1.001+0.172i for tan δ = 0.12), resulting in smaller reflectance and then higher absorbance.

As for the broadband response of the double-sided MPA, we attributed this broadband characteristic to two possible reasons; one is the quality factor of the MPA and the other is the mergence of two resonance peaks. To examine the first reason, the quality factor, we should scrutinize Fig. S1(c) again and observe that the bandwidth of absorbance becomes much broader as tan δ of PET increases, originating from a lower quality factor based on $Q = \frac{f_0}{FWHM}$. Also, in Fig. S1(b), the reflectance spectrum with loss-free PET reveals that there coexist two reflectance dips, developing minor and major absorbance bands at 2.30 THz and 2.40 THz, respectively. Once loss tangent of PET becomes higher and higher, these two absorbance peaks appear broader and broader and finally merging together, resulting in a broadband absorbance for the case of tan δ = 0.12 in our work.

- ## Thermal losses of the double-sided MPA

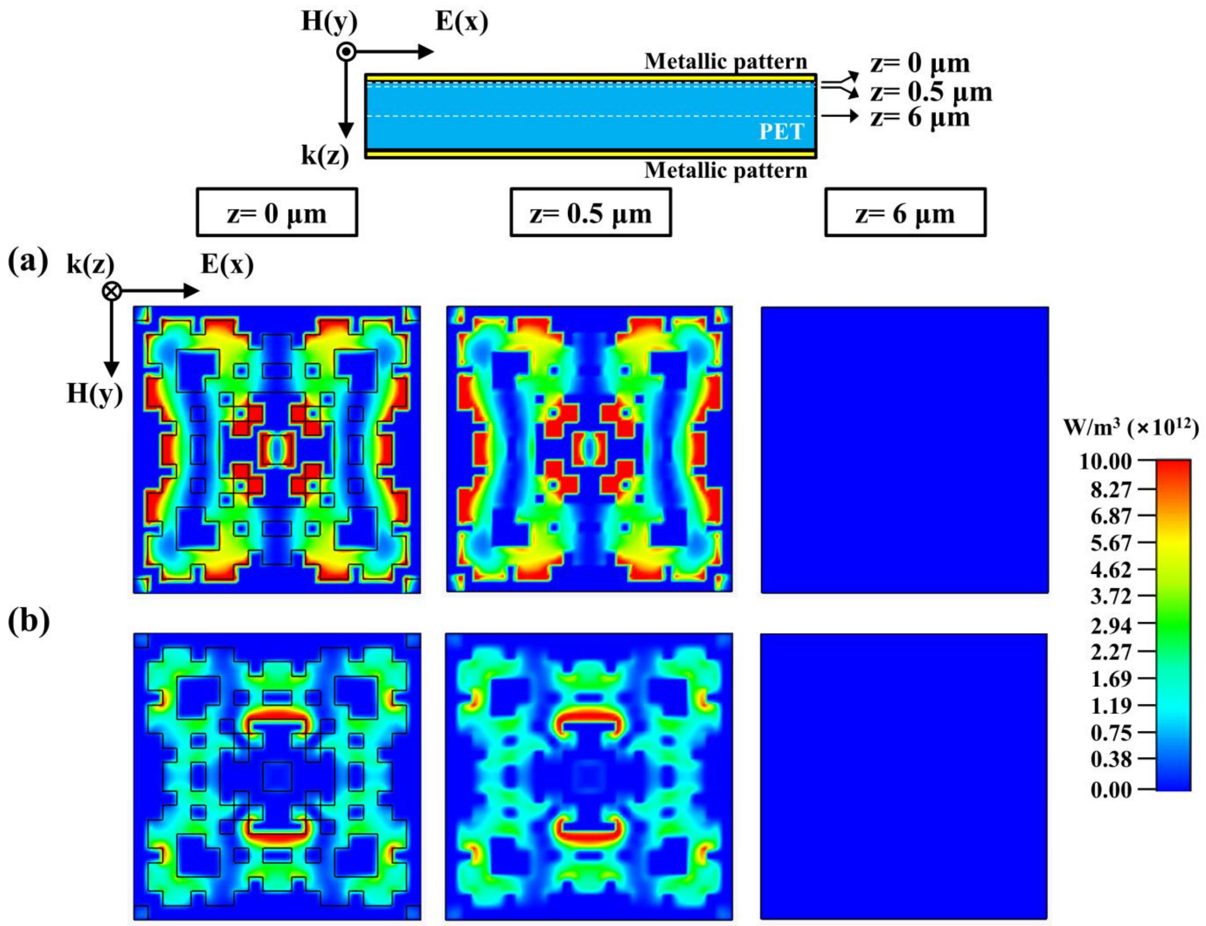

Figure S3. (a) Thermal volume loss and (b) thermal surface loss distribution within the double-sided MPA with respect to x-y view. As expected, away from the metallic structure, there appear few thermal losses within PET.

We calculated the corresponding volume loss $P_v = \pi f tan\delta \varepsilon_0 \varepsilon_r \int |E|^2 dV$, and surface loss $P_s = \frac{1}{2}\sqrt{\frac{\pi \mu f}{\sigma}} \int |H|^2 dS$, respectively (i.e., total loss power $P = P_v + P_s$). As expected, the thermal losses mainly concentrate around the metallic patterns and are gradually decreasing away from metal due to higher conductivity of metal compared to the dielectric spacer as shown in Fig. S3.

It is worth mentioning once we insert a layer of an energy reservoir such as thin film solar cells within the spacer[S1], hence electromagnetic energy trapped by our absorber could be transformed into electricity and heat dissipation would be no longer the only route for

energy conversion. This is why we only put the field distribution in the main content and claim that most fields are confined within the spacer region.

• Thickness-dependent response of the MPA

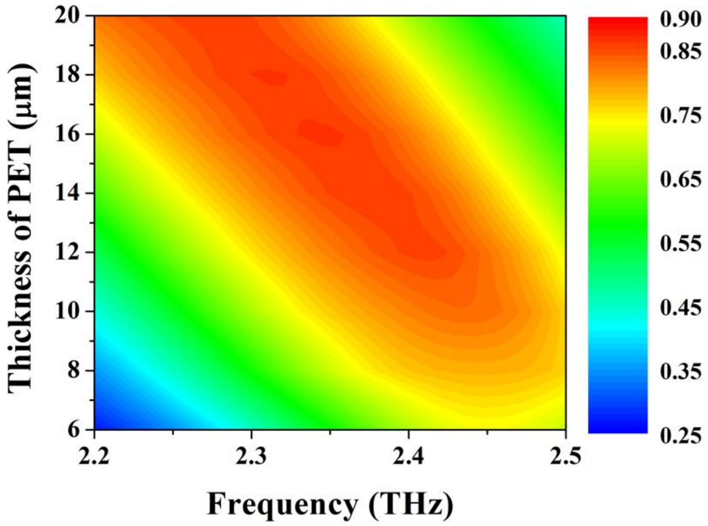

Figure S4. Thickness dependence of the double-sided MPA. An obvious red shift is observed due to increases of an optical path with increasing thicknesses of PET. Such optical path changes contribute to a significant and a minor decrease of maximum absorbance as thicknesses of PET are thinner and greater than 12 μm, respectively.

• Angle-dependent response of the MPA

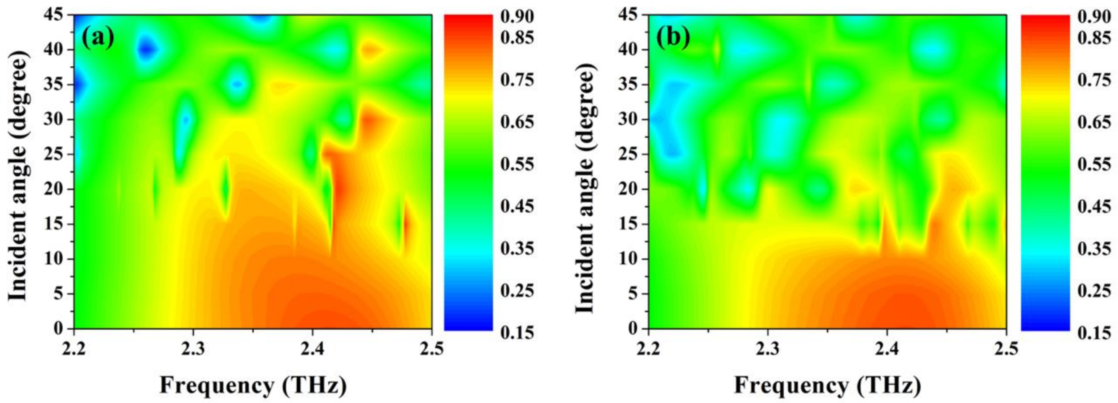

Figure S5. Angle-dependence of the double-sided MPA for (a) TE and (b) TM cases. Our double-sided MPA could tolerate oblique incident angles up to around 10-degree. After this angle, the absorbance band becomes split with smaller absorbance peaks for both TE and TM cases.

Reference:

S1. Wang, Y. *et al.* Metamaterial-Plasmonic Absorber Structure for High Efficiency Amorphous Silicon Solar Cells. *Nano Lett.* **12**, 440–445 (2012).
